# Supplementary material for: Understanding spatial effects in species distribution models
Source: PLoS One. 2023 May 30;18(5):e0285463. doi: 10.1371/journal.pone.0285463 (PMC10228761; doi:10.1371/journal.pone.0285463)
Supplement: S1 File — (ZIP) [file pone.0285463.s001.zip › Supporting information.pdf]

Understanding spatial effects in species distribution models

Iosu Paradinas[1,2*], Janine Illian[2], Sophie Smout[2,3]

1 Scottish Ocean's Institute. University of St Andrews. East sands, St Andrews, UK.
2 AZTI, Txatxarramendi Ugartea z/g, 48395, Sukarrieta, Bizkaia, Spain
3 School of Mathematics and Statistics, University of Glasgow, Glasgow, G12 8QQ, UK

* ip30@st-andrews.ac.uk

1 Supporting information

This document explains the procedure that we followed to create the Table 2 of the manuscript. To do so we use a single simulated species distribution (as compared to 50 simulations in the study) that is also driven by three spatially structured environmental covariates acting at different spatial scales and a geographical range dispersion process. We fitted all the models described in the manuscript and we computed SIP scores between each model's spatial effect and all the possible different combinations of covariate surfaces. By doing so, we get Table 1, which displays highest SIP scores along the diagonal, matching the combination of drivers (columns) with the covariates that are missing in the fitted models (rows).

	Combination of drivers							
Model	Dispersion	S	M	L	S & M	S & L	M & L	S, M & L
M_0	-0.01	0.43	0.57	0.49	0.71	0.55	0.73	**0.82**
M_S	0.12	-0.04	0.73	0.43	0.49	0.25	**0.85**	0.59
M_M	0.03	0.50	0.19	0.69	0.47	**0.72**	0.46	0.72
M_L	0.05	0.40	0.75	0.06	**0.78**	0.33	0.65	0.75
M_SM	0.16	0.03	-0.09	**0.81**	-0.03	0.48	0.46	0.33
M_SL	0.11	-0.06	**0.83**	-0.15	0.57	0.01	0.76	0.53
M_ML	0.01	**0.64**	0.13	0.03	0.57	0.54	0.05	0.57
M_SML	**0.22**	-0.05	0.17	-0.12	-0.00	-0.12	0.10	0.09

Table 1. SIP scores between fitted spatial effects and all the combinations of covariate surfaces. Scores must be read by row. Values closer to one reflect bigger resemblance between spatial fields.

Once we repeat the simulation 50 times we get 50 SIP scores for each position in the table, which could be summarised by the mean and standard deviation of these 50 values. However, we decided to use the difference between the best SIP score for each model and combinations of covariate fields because results were clearer, i.e. differences by row in the Table 1. This way Table 1 becomes Table 2, where zero values represents the best SIP score per model (by row) and the rest of the scores represent the SIP score difference with respect to the best score by row.

	Combination of drivers							
Model	Dispersion	S	M	L	S & M	S & L	M & L	S, M & L
M_0	0.83	0.39	0.24	0.32	0.11	0.27	0.08	**0.00**
M_S	0.74	0.89	0.13	0.43	0.36	0.60	**0.00**	0.26
M_M	0.70	0.23	0.53	0.03	0.26	**0.00**	0.26	0.01
M_L	0.74	0.38	0.03	0.72	**0.00**	0.45	0.13	0.03
M_SM	0.66	0.78	0.90	**0.00**	0.85	0.33	0.35	0.48
M_SL	0.72	0.90	**0.00**	0.99	0.26	0.82	0.07	0.30
M_ML	0.63	**0.00**	0.51	0.61	0.06	0.10	0.59	0.07
M_SML	**0.00**	0.28	0.05	0.34	0.23	0.35	0.13	0.13

Table 2. The difference in score between the best SIP score and the rest for each model (by row). Values closer to zero reflect bigger resemblance between spatial fields.
