# Supplementary material for: Understanding spatial effects in species distribution models
Source: PLoS One. 2023 May 30;18(5):e0285463. doi: 10.1371/journal.pone.0285463 (PMC10228761; doi:10.1371/journal.pone.0285463)
Supplement: Attachment — Submitted filename: Review PONE-D-22-21589.docx [file pone.0285463.s002.docx]

**Review: Understanding spatial effects in species distribution models**

**Summary:**

Paradinas et al., present a simulation study to assess how random spatial effects represent missing co-variates using two spatial models: a Generalised Additive Model (GAM) and a Bayesian spatial model (INLA). They demonstrate that fitted spatial effects resemble the sum of the unaccounted covariate surfaces in each model.

The simulation approach is relatively simple (as acknowledged by the authors in the discussion) but clearly explained and relevant for the aims of the study. I have no major concerns with this study, however, I think the manuscript could benefit from some additional points in the introduction and discussion in order to further appeal to ecologists who may want to consider the results from this interesting study (see specific comments below).

**Abstract & general comments to consider throughout the text**

I’m not sure I agree with “most SDMs include spatial effects”. Consider softening with “SDMs can account for random spatial effects which may improve prediction… etc”. Note that the use of random spatial effects is sometimes used in linear modelling (Bayesian or not) but less so for machine learning which are popular SDM algorithms (e.g., Random Forest, Boosted Regression Trees, Maxent, etc) – you could consider including this point somewhere in the MS – possibly in the discussion?

Note in my suggested wording above, I use the term “random spatial effects”. If the authors agree with this clarification. Suggest this term is used throughout the text, or at first mention to make clear.

I’m assuming that throughout that the manuscript the authors are referring to SDMs fitted with occurrence data (the most common SDMs in my experience). If so, please make this very clear early on. If not, then further explanation is needed for how to interpret statements about Type I error for SDMs fitted with occurrence data AND with those fitted with abundance data (since for the latter, you may need to approach this as a hurdle model or delta model).

The authors refer to “Type I errors”. I don’t disagree that this term is an accurate description, but I think many ecologists in the context of SDMs (where the null hypothesis is not clear) would have difficulty interpreting the statement. For example, if the authors are referring to SDM fitted with occurrence data, would type I refer to “false positive rate” and “false negative rate” i.e., misclassification of presences (predicted 0 when it should 1) and misclassification of absences (predicted 1 when it should 0)? Can you try and link this explanation to commonly used model fit metrics for SDMs such as TSS (True Skill Statistic) which specifically deal with sensitivity and specificity?

Again, I’m not sure I agree with the statement “Ecologists tend to try ecologically interpret the spatial patterns displayed by the spatial effect” nsuggest softening it to “In some cases ecologists try to ecologically interpret the spatial patterns displayed by the spatial effect” since some SDM studies are principally concerned with predictive ability rather than interpretation of drivers.

Suggest instead of “This study wants to provide a practical demonstration” the authors use “This study aims to provide a practical demonstration”

Suggest that the authors generalise the description of the methods to “Generalised Additive Model” and shortened to “GAM” and “Bayesian spatial model” when generally referring to the methods in the intro / discussion – in my experience this terminology is more informative / commonly used than “2D smoothing splines” and “geostatistic model” respectively.

**Introduction**:

L 8-10: I think the authors need to broaden this out to link this more with ecology. Suggest rejigging this sentence to reflect the below points

1. ideally co-variates (spatial and non-spatial) would fully explain the relationship between species occurrence or abundance with biotic and abiotic factors (you could briefly explain this in the context of ecological niches?).
2. In practice this isn’t possible since it’s really complicated (you mention this in the discussion) and the co-variates we have aren’t always accurate.
3. Random spatial or spatial/temporal effects can be incorporated in SDMs to **proxy for** unaccounted for processes which have a spatial, temporal, spatial-temporal structure (i.e., missing co-variates / unaccounted for interactions between co-variates).

L8 – 9: Can you also provide examples of each co-variate in brackets? E.g., assuming that “non-spatial covariates” would be things like year, month?

L14 -16: my above point about proxying for unaccounted for co-variates is relevant here. I agree with the authors but suggest that you clarify that these are due to “unaccounted environmental or biotic drivers” which are often variables which are hard to measure or estimate, e.g., “geographical range dispersion process, e.g. colonisation and other highly dynamic processes such as wind and current”.

L17 – 23: very good points about scale

L43: suggest remove “novel”

L46: suggest remove “rather”

L47: I’m not familiar with the terminology “model-based spatial models” Can you not simply call these “spatial models”? Or simplify some other way?

**Simulation:**

Suggest that you need to clarify somewhere in this section:

- whether your SDMs are occurrence
- that you calculated the residuals (table 2) and what these represent (i.e., deviations predicted from actual empirical values of data – which comes back to the units need to be clarified)

L50: depending on the formatting requirements of the journal, this may be better called “methods”

L52: this is a good example of an often-omitted co-variate which random spatial effects are often interpreted as representing. Suggest you (briefly) revisit this in the discussion.

L64-66: see suggestions on model terminology. Also need to state whether you used default settings (which I assume you did).

**Results**:

L93 – 95: suggest this is discussion rather than results.

**Discussion**:

L99: suggest need a reference as an example for this statement

L104 -105: suggest, “Here, we did not attempt to account for all possible cases, instead, we aimed to illustrate our point using a simple and intuitive approach.”

L124 – 126: Agree that this can increase predictive capacity, but only within sampled areas (i.e., interpolation between sample points). Although extrapolation isn’t encouraged, in many cases SDMs are spatially predicted into areas with low samples where random spatial effects provide no additional benefit (especially if they override the effects of other co-variates which are important drivers). I think you could mention this caveat. That is, to use spatial effects with care when the aim if prediction.
